# The Effects of Intravenous Immunoglobulins in Women with Recurrent Miscarriages: A Systematic Review of Randomised Trials with Meta-Analyses and Trial Sequential Analyses Including Individual Patient Data

**DOI:** 10.1371/journal.pone.0141588

**Published:** 2015-10-30

**Authors:** Pia Egerup, Jane Lindschou, Christian Gluud, Ole Bjarne Christiansen, ImmuReM IPD Study Group

**Affiliations:** 1 The Fertility Department, Rigshospitalet, Copenhagen University Hospital, Copenhagen, Denmark; 2 The Copenhagen Trial Unit, Centre for Clinical Intervention Research, Rigshospitalet, Copenhagen University Hospital, Copenhagen, Denmark; 3 The Fertility Department, Rigshospitalet, Copenhagen University Hospital, Copenhagen, Denmark, Copenhagen Trial Unit, Centre for Clinical Intervention Research, Rigshospitalet, Copenhagen University Hospital, Copenhagen, Denmark, Department of Obstetrics and Gynaecology, University Hospital Linköping, Linköping, Sweden; 4 Department of Obstetrics and Gynaecology, Aalborg University Hospital, Aalborg, Denmark; University of Ottawa, CANADA

## Abstract

**Background:**

Immunological disturbances are hypothesised to play a role in recurrent miscarriage (RM) and therefore intravenous immunoglubulins (IVIg) have been tested in RM patients.

**Objectives:**

The objectives were to investigate the benefits and harms of IVIg versus placebo, no intervention, or treatment as usual in women with RM.

**Search Strategy:**

We searched the published literature in all relevant databases.

**Selection Criteria:**

Randomised trials investigating IVIg versus placebo, no intervention, or treatment as usual in women with RM.

**Data Collection and Analysis:**

We undertook meta-analyses of aggregated data and individual patient data using a two-step approach, and we conducted bias domain assessments and trial sequential analyses to assess the risks of systematic and random errors.

**Main Results:**

We identified 11 randomised clinical trials. No significant difference in the frequency of *no live birth* was found when IVIg was compared with placebo or treatment as usual (RR 0.92, 95% CI 0.75–1.12, p = 0.42). Trial sequential analysis showed that the required information size of 1,008 participants was not obtained. IVIg compared with placebo seems to increase the risk of adverse events. Subgroup analysis suggests that women with RM after a birth (secondary RM) seemed most likely to obtain a potential beneficial effect of IVIg (RR for *no live birth* 0.77, 95%CI 0.58–1.02, p = 0.06), however, trial sequential analysis showed that insufficient information is presently accrued.

**Conclusion:**

We cannot recommend or refute IVIg in women with RM. IVIg should therefore be assessed in further randomised clinical trials with positive outcomes before any clinical use is considered.

## Introduction

Recurrent miscarriage (RM) is generally defined as three or more miscarriages before gestational week 24 [1]. Many clinicians define, however, RM as two or more miscarriages [1]. Primary RM refers to a series of miscarriages without a previous live birth whereas secondary RM refers to a series of miscarriages subsequent to a previous birth. Some clinicians also use the term secondary RM if the miscarriages have been preceded by a live birth or stillbirth after gestational week 22 [2]. RM affects 1% to 5% of all women trying to conceive [1]. In only a minority can the condition be explained by parental chromosomal abnormalities, uterine malformations, infective causes, or endocrine or thrombophilic disturbances [1].

Immunological disturbances are hypothesised to play an important role in RM and therefore various types of immune-based interventions have been tested in RM patients, including intravenous immunoglobulins (IVIg) [3–5].

IVIg have several effects like suppression and neutralisation of autoantibodies, attenuation of natural killer cells, inhibition of complement binding, modification of cytokine production, and expansion of regulatory T lymphocytes [6, 7]. IVIg exhibit a documented effect in many disorders caused by immunological abnormalities [8]. IVIg formulations are made by extracting the IgG fractions from plasma from normal blood donors and therefore there are potential risks of adverse events like allergy and transmission of infections (e.g., HIV, hepatitis). In general, IVIg are well tolerated, and the most frequent adverse reactions, which include headache, fever, and nausea, occur in less than 5% of patients [6].

So far, several randomised placebo-controlled trials investigating IVIg in women with RM have been published with conflicting results [5, 9–15].

The newest systematic review with meta-analysis [16] was updated in February 2014 and found overall no significant beneficial effect of IVIg versus placebo in improving the live birth proportion. However, this review only assessed one outcome (birth after 20 weeks of gestation) and a single subgroup analysis, it did not include a randomised placebo-controlled trial of IVIg in RM treatment published in 2010 [15], it did not assess the risk of random errors, and it did not adequately investigate the full effect of benefits or harms of IVIg.

Due to the recent publication of a new randomised trial [17] and the considerations above, we found it relevant to conduct an up-dated systematic review with meta-analysis and trial sequential analyses (TSA) of randomised trials of IVIg versus placebo, no intervention, or treatment as usual in women with RM. Furthermore, we included individual patient data (IPD) in the meta-analyses whenever possible. IPD allow data checking and re-analysis of data in a consistent way. It also allows categorisation of participants in order to conduct subgroup analyses not feasible using aggregate data. Subgroup analysis is clinically relevant since it allows a more personalised treatment [18–20].

## Methods

This systematic review was conducted according to our published protocol [21]. The protocol was registered within the International Prospective Register of Systematic Reviews (PROSPERO) as number CRD42014007112.

### Search strategy

We searched the relevant published literature using the following databases: the Cochrane Central Register of Controlled Trials (Central December 2014), Medline (1950 to December 2014), Embase (1947 to December 2014), WHO International Clinical Trials Registry Platform (December 2014), and Ovid Medline In-Process and Other Non-Indexed Citations databases (December 2014). The following medical subject headings (MeSH) terms, keywords, and their combinations were used: immunoglobulins; intravenous; immunotherapy; foetal death, abortion; habitual abortion; spontaneous; foetal loss; miscarriage; recurrent abortion; recurrent miscarriage. Appropriate suffixes were used for each database. The Cochrane Collaboration strategy for identifying randomised trials using the relevant MeSH terms and keywords were used. Similar search strategies were used in Central, Embase and Ovid Medline In-Process and other non-indexed citation databases. Relevant abstracts from the annual meetings of American and European Societies of Reproductive Medicine and Human Reproduction were searched. The reference lists of the identified reports were manually searched for other relevant publications. The full search strategy can be seen in S2 and S3 Files.

No language restrictions were applied.

### Selection of trials and data extraction

All randomised trials irrespective of publication date, publication type, publication language, and publication status investigating infusions with IVIg in relation to pregnancy in women with RM compared with placebo, no intervention, or treatment as usual were included for assessments of benefits and harms. For assessments of harms, quasi-randomised clinical studies and observational studies that we identified during our search for randomised clinical trials were included.

We used the instructions in The Cochrane Handbook for Systematic Reviews of Interventions [22] and The Cochrane Hepato-Biliary Group Module [23] in our evaluation of the methodology and hence bias risk of the included trials [24–29]. A full description on risk of bias assessment can be found in our published protocol [21].

Two authors (PE and JL) independently identified trials for inclusion, assessed risk of bias, and extracted data from all included trials. The first authors of the original articles were contacted by e-mail with the aim of collecting IPD. If the first e-mail was not answered, a new e-mail was sent out one month later. If the second e-mail was not answered, we tried to contact some of the co-authors of the trial. The original authors were invited to become part of the ImmuReM (Intravenous **Immu**noglobulins in **Re**current **M**iscarriage) IPD Study Group and thereby co-authors of the present article, provided they delivered IPD.

### Outcomes

#### Primary outcomes

1. The proportion of women not giving live birth, defined according to the trialists. It is usual practise in Cochrane to choose a negative outcome. This is because RR can be interpreted the same way if all outcomes are negative.
2. The proportion of women experiencing a serious adverse event (SAE) defined as any adverse event that results in death, is life-threatening, requires hospitalisation or prolongation of existing hospitalisation, or results in persistent or significant disability or incapacity [30]. SAEs were assessed as a composite of all the above events.
3. The proportion of live-born infants experiencing SAEs defined as any adverse event that results in death, is life-threatening, requires hospitalisation in a neonatal care unit or prolongation of existing hospitalisation, results in persistent or significant disability or incapacity or is a congenital anomaly or birth defect [30]. SAEs were assessed as a composite of all the above events.

#### Secondary outcomes

1. The proportion of women experiencing an adverse event (AE) defined as any undesirable medical event occurring to a participant during a clinical trial, which does not necessarily have a causal relationship with the intervention [30]. AEs were assessed as a composite of all the above events.
2. The women’s quality of life, as defined by the trialists.
  The secondary outcomes for women with a live birth in the trial and their infants were:
3. Proportion of women who gives live birth prematurely (<37 weeks).
4. Sex of the infant.
5. The proportion of infants with low birth weight, i.e., <2500 g.
6. The proportion of infants experiencing AEs. AEs were assessed as a composite.
7. The infants’ quality of life, as defined by the trialists.

### Data synthesis

We undertook meta-analyses according to the recommendations of The Cochrane Handbook for Systematic Reviews of Interventions [22]. For binary outcomes we calculated a standard estimation of the risk ratio (RR) and its 95% confidence interval (CI). For all outcomes and according to availability, we combined IPD and published aggregate data into a pooled effect measure using a two stage method [20]. First, IPD for a given trial were analysed to construct summary data, second the summary data were added to the meta-analysis along with published aggregated data from trials without IPD data [20].

We applied Trial Sequential Analysis (TSA) because cumulative meta-analyses are at risk of producing random errors as the result of sparse data or repetitive testing of accumulating data [31]. TSA can provide a required information size, a threshold for a statistically significant treatment effect (benefit or harm), and a threshold for futility [31]. The required information size indicates the number of patients needed to detect or reject a certain intervention effect. TSA makes it possible to test for statistical significance before the required information size has been reached. The trial sequential monitoring boundaries determine the statistical inference that one may draw regarding a cumulative meta-analysis that have not reached the required information size; if the cumulative Z-cure of the meta-analysis crosses a trial sequential boundary for benefit or harm before the required information size is reached, firm evidence may be established, and further trials may turn out to be superfluous. Futility boundaries are a set of thresholds that reflects the uncertainty of obtaining a negative finding in relation to the strength of the available evidence (e.g., the accrued number of patients). Below the threshold, the test statistic is so low that the likelihood of a significant effect being found if one reaches the required information size becomes negligible. On the other hand, if futility boundaries are not crossed, it is most probably necessary to continue doing trials to detect or reject a plausible intervention effect [31].

We performed TSA on the primary outcomes, in order to control the risks of type I and type II errors that occur in traditional meta-analyses due to sparse data and repetitive analyses of accumulating data [31–36]. In order to control these risks, we calculated the diversity-adjusted required information size and assessed the eventual breach of the cumulative Z-curve of the relevant trial sequential monitoring boundaries [31]. The calculation was based upon the proportion with the outcome in the control group; a relative risk reduction of 20%; an alpha of 5%; a beta of 20%, and the diversity (D^2^) of the meta-analysis assessed by the TSA programme [37].

## Results

### Results of the searches

**Fig 1:**
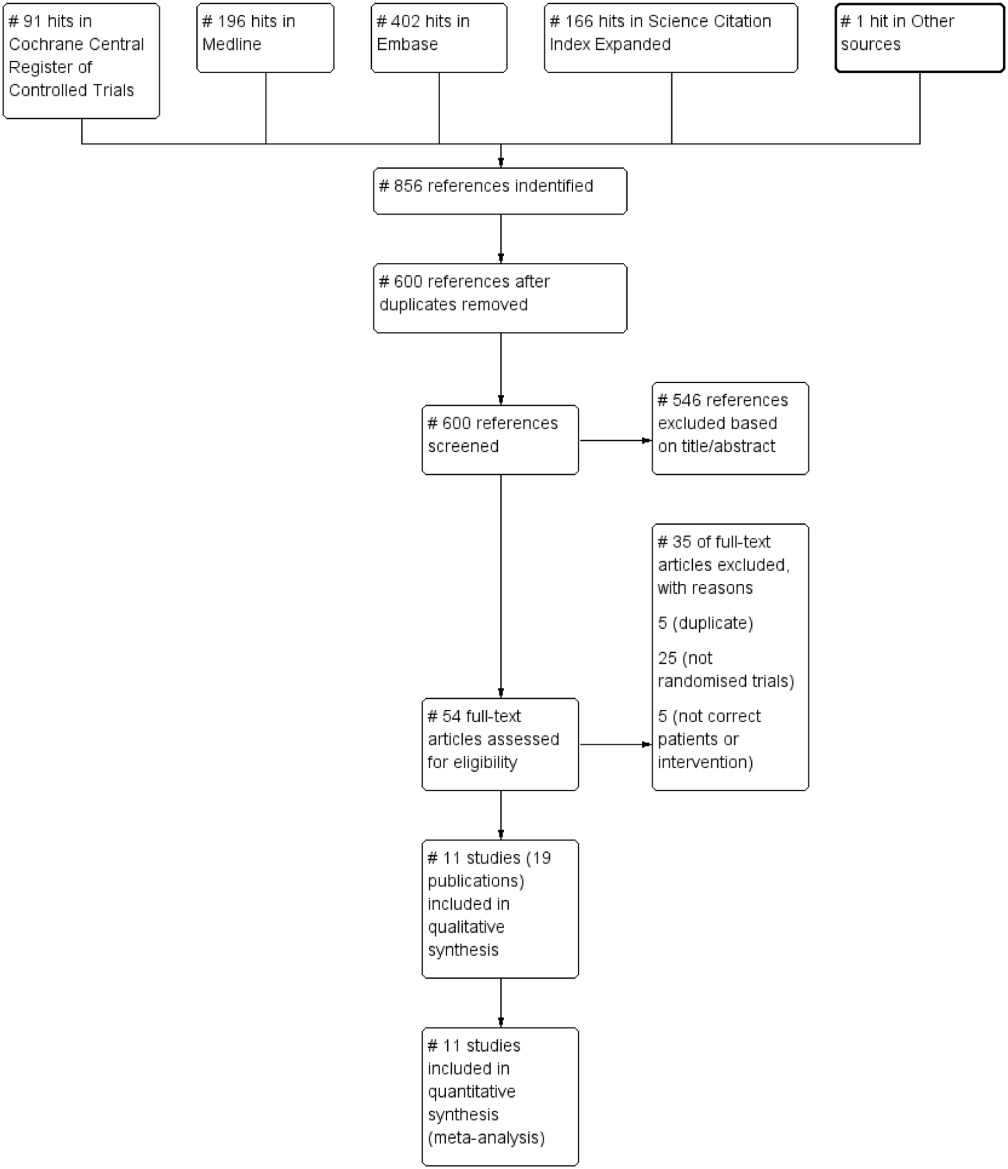
PRISMA flow diagram illustrating the selection procedure.

Our predefined searches 01-12-2014 identified 856 references, including 256 duplicates. The PRISMA flow diagram illustrating the selection procedure is shown in Fig 1.

Of the 600 remaining individual references, 546 were excluded based on title and abstract. After reviewing full-text articles of the remaining 54 references, 35 were excluded. Reasons for exclusions were that publications were duplicates, clinical trials were not randomised, or the patients or interventions were not correct according to our inclusion criteria.

We included 11 randomised clinical trials described in 19 publications in our systematic review [5, 9–15, 17, 38, 39].

We identified four observational studies, but no quasi-randomised studies during our searches [40–43]. The observational studies were included for assessments of harms.

### Included trials

Table 1 shows the baseline characteristics of the 11 included trials.

**Table 1:** Baseline characteristics of the included trials. Baseline characteristics of the included trials according to IVIg regimen, placebo participants and types of miscarriage.

Authors	IVIg regimen	Placebo	Participants	Types of recurrent miscarriage
***The German RSA/IVIG Group, 1994 [11]***	IVIg 5%, 30 g when pregnancy was detected, then 20 g every 3 weeks until week 25.	Human albumin 5%, given in the same regimen as IVIg	65 women were randomised and included.IVIg: 33Placebo: 32	Primary RM.Minimum 3 previous miscarriages.
***Christiansen et al., 1995 [10]***	Nordimmun^®^ (containing IVIg, human albumin and saccharose). The infusions were started immediately after pregnancy was detected. Women <60 kg: a total of 380 g. Women 60–80 kg: a total of 465g. Women >80 kg: a total of 550 g. A total of 17 infusions were given in successful pregnancies until week 34	Human albumin and saccharose given in the same regimen as IVIg.	34 women were randomised and includedIVIg: 17Placebo: 17	Secondary RM or women with RM and second trimester losses.Minimum 3 previous miscarriages.
***Coulam et a.l, 1995 [9]***	IVIg, 500 mg/kg per month. Each patient received an infusion in the follicular phase of the cycle, when pregnancy was desired. Once conception occurred, the patient received an infusion every 28 days until delivery or 28–32 week.	0.5% albumin given in the same regimen as IVIg.	61 women were randomised and achieved pregnancy.IVIg: 29Placebo: 32	Both primary and secondary RM. Two or more consecutive miscarriages
***Perino et al., 1997 [12]***	25 g IVIg /day on two consecutive days (weeks 5 to 7) when the pregnancy was diagnosed. A third dose of 25 g was administrated 3 weeks later when ultrasound scanning confirmed an ongoing pregnancy.	Saline solution with 5% human albumin given in the same regimen as IVIg.	46 women were randomised. IVIg: 22Placebo: 24	Primary RM. Minimum 3 previous miscarriages.
***Stephenson et al., 1998 [13]***	Gamimune N^®^ (5% IVIg in normal saline at a dose of 500mg/kg). The initial infusion was given in the follicular phase of the menstrual cycle in which the woman attempted to achieve pregnancy. Total number or amounts of infusions are not reported.	Saline infusions given in the same regimen as IVIg.	39 women were randomised and became pregnant. IVIg: 20. Placebo: 19.	Both primary and secondary RM.Minimum 2 previous miscarriages.
***Jablonowska et al., 1999 [14]***	IVIg (20 g Gammonativ^®^, 400 ml) was given every three weeks on five occasions if a viable pregnancy was confirmed by ultrasound before each treatment. The intervention was started at gestational weeks 6–8, when foetal heart activity was diagnosed by ultrasound.	Saline infusions (400 ml) given in the same regimen as IVIg.	41 women were randomised. IVIg: 22. Placebo: 19	Both primary and secondary RM.Minimum 3 previous miscarriages.
***Christiansen et al., 2002 [5]***	Nordimmun^®^ (4.6% human IgG, 4.6% sucrose, 1.5% human albumin and 0.15 mol/l sodium). The first infusion was given after a positive pregnancy test in week 5. Weekly infusions until week 10, then every second week until week 26.0.8 g IVIg/kg until week 20 and then 1.0 g IVIg/kg.	The placebo drug contained 1.5% human albumin, 4.6% sucrose and 0.15 mol/l sodium and was given in the same regimes as IVIg.	58 women were randomised.IVIg: 29Placebo: 29	Both primary and secondary RM.Minimum 4 previous miscarriages.
***Mahmoud et al., 2002 [39]***	500 mg IVIg/kg/monthThe authors report a regimen of 0.5 mg/kg bodyweight daily for 5 days every month until about 34 week.	Multivitamins, not otherwise defined.	15 women were randomised. IVIg: 7 Control: 8.	Primary and secondary RM. Minimum 3 previous miscarriages.
***Triolo et al., 2003 [38]***	IVIg (IgVENA N, Sclavo, Siena, Italy). Dose 400 mg/kg/day for two consecutive days after a positive pregnancy test followed by a single dose each month until week 31 or at the time of miscarriage.	Low molecular weight (LMW) heparin, self-administered subcutaneous injection of 5,700 IU daily plus low-dose aspirin, 75 mg daily. Aspirin was discontinued at 34 weeks of gestation and heparin at 37 weeks of gestation.	42 women were randomised.IVIg: 21.Heparin + aspirin: 21	Minimum 3 previous miscarriages.
***Stephenson et al., 2010 [15]***	Gamimune^®^ or Gamunex^®^ at a dose of 500 mg/kg. Preconception infusions were administered 14–21 days from the projected next menstrual period. With documentation of pregnancy similar infusions were given every 4 weeks until 18–20 weeks.	Saline infusions giving in the same regimen as IVIg.	77 women were randomised. 24 in the IVIg group and 28 in the placebo group became pregnant and completed the intervention	Only secondary RM.Minimum 3 previous miscarriages.
***Christiansen et al., 2014 [17]***	Immunoglobulin human CSL Behring^®^ 120 mg/ml or Privigen^®^ 100 mg/ml. For participants with weight <75 kg, 24 g / 25 g and for those weighing ≥75 kg, 36 g / 35 g was given at each infusion. The first infusion was given as soon as pregnancy was diagnosed by plasma hCG measurements. In ongoing pregnanies a total of 8 infusions were given until week 15.	200 ml / 250 ml or 300 ml / 350 ml human albumin CSL Behring^®^ 5%, respectively, was given at each infusion according to the weight groups.	82 women were randomised. IVIg: 42. Placebo: 40	Only secondary RM.Minimum 4 previous miscarriages.

Two trials enrolled only women with primary RM [11, 12], two trials enrolled only women with secondary RM [15, 17], and six trials enrolled women with primary or secondary RM [5, 9, 10, 13, 14, 39]. One trial did not report whether the women had primary or secondary RM [38]. The experimental intervention was IVIg in all trials. Dosage and number of infusions varied between trials. Eight trials initiated the intervention when the pregnancy was detected by a positive pregnancy test or by ultrasound [5, 10–12, 14, 17, 38, 39] and three trials initiated the intervention before the women became pregnant [9, 13, 15].

Six trials used albumin as placebo [5, 9–12, 17] and three trials used normal saline as placebo [13–15]. Albumin is considered an adequate placebo for IVIg as IVIg and albumin cannot be distinguished if the vials are identical. Saline is not considered an adequate placebo as it can be distinguished visually from the IVIg. Two trials used treatment as usual with multivitamin and subcutaneous heparin plus low-dose aspirin, respectively, as control interventions [38, 39]. These two control interventions could easily be distinguished from IVIg as they are applied differently.

IPD were obtained from four trials, including Jablonowska et al. [14], Christiansen et al. [5] Christiansen et al. [10], and Christiansen et al.[17]. From the trials by Perino et al. [12] and Stephenson et al. [13] some of the IPD could be extracted directly from the publications.

### Risk of bias

Table 2 illustrates risk of bias in all the included trials.

**Table 2:** Risk of bias in the included trials. Risk of bias in the included trials according to a series of domains. In relation to these assessment of these domains the trials were classified as high or low overall risk of bias.

Authors	Allocation sequence	Allocation concealment	Blinding of the participants and treatment providers	Blinding of outcome assessors	Incomplete outcome data	Selective outcome reporting	Forprofit bias	Other souces of bias	Overall risk of bias
***The German RSA/IVIG Group, 1994 [11]***	Low*	Low	Low	Unclear^†^	Low	Unclear	High^‡^	Low	High (Low)^§^
***Christiansen et al., 1995 [10]***	Low	Low	Low	Low	Low	Unclear	High	Low	High (Low)
***Coulam et al., 1995 [9]***	Low	Unclear	Low	Unclear	Low	Unclear	Unclear	Low	High
***Perino et al., 1997 [12]***	Low	Low	Low	Unclear	Low	High	High	Low	High (Low)
***Stephenson et al., 1998 [13]***	Unclear	Low	Unclear	Unclear	Low	Unclear	Low	Low	High
***Jablonowska et al, 1999 [14]***	Unclear	Low	Unclear	Unclear	Low	Unclear	High	Low	High
***Christiansen et al., 2002 [5]***	Low	Low	Low	Low	Low	Unclear	High	Low	High (Low)
***Mahmoud et al., 2002 [39]***	Unclear	Unclear	High	Unclear	Unclear	Unclear	Unclear	Low	High
***Triolo et al., 2003 [38]***	Low	Low	High	Unclear	Low	Unclear	Unclear	Low	High
***Stephenson et a.l, 2010 [15]***	Low	Low	Unclear	Unclear	Low	Unclear	High	Low	High
***Christiansen et al., 2014 [17]***	Low	Low	Low	Low	Low	Low	Low	Low	Low

* Low: Low risk of bias: that is the domain is likely not associated with bias (i.e., overestimation of benefits and underestimation of harms).

^†^ Unclear risk of bias: it is unclear if the domain is associated with risk of bias (i.e., overestimation of benefits and underestimation of harms).

^‡^ High risk of bias: that is the domain is likely associated with risk of bias (i.e. overestimation of benefits and underestimation of harms).

^§^ (Low) risk of bias overall: low risk of bias in the selected domains: allocation sequence, allocation concealment, and blinding of participants and treatment providers. Accordingly, these trials could still be biased due to other domains.

Only one trial [17] was classified as ‘low risk of bias’ and ten trials were classified as ‘high risk of bias’. Of the trials with ‘high risk of bias’, four trials were classified as ‘low risk of bias’ according to selected domains: allocation sequence, allocation concealment, and blinding of participants and treatment providers [5, 10–12]. Such trials can, however, be biased by other domains.

All meta-analyses were performed both with a fixed-effect and a random-effects model. The following results are based on the random-effects model; however, the fixed-effect model provided similar results.

We have combined aggregate data and IPD in all our meta-analyses by using a two stage method [20, 44]

### The proportion of women not giving live birth

**Fig 2:**
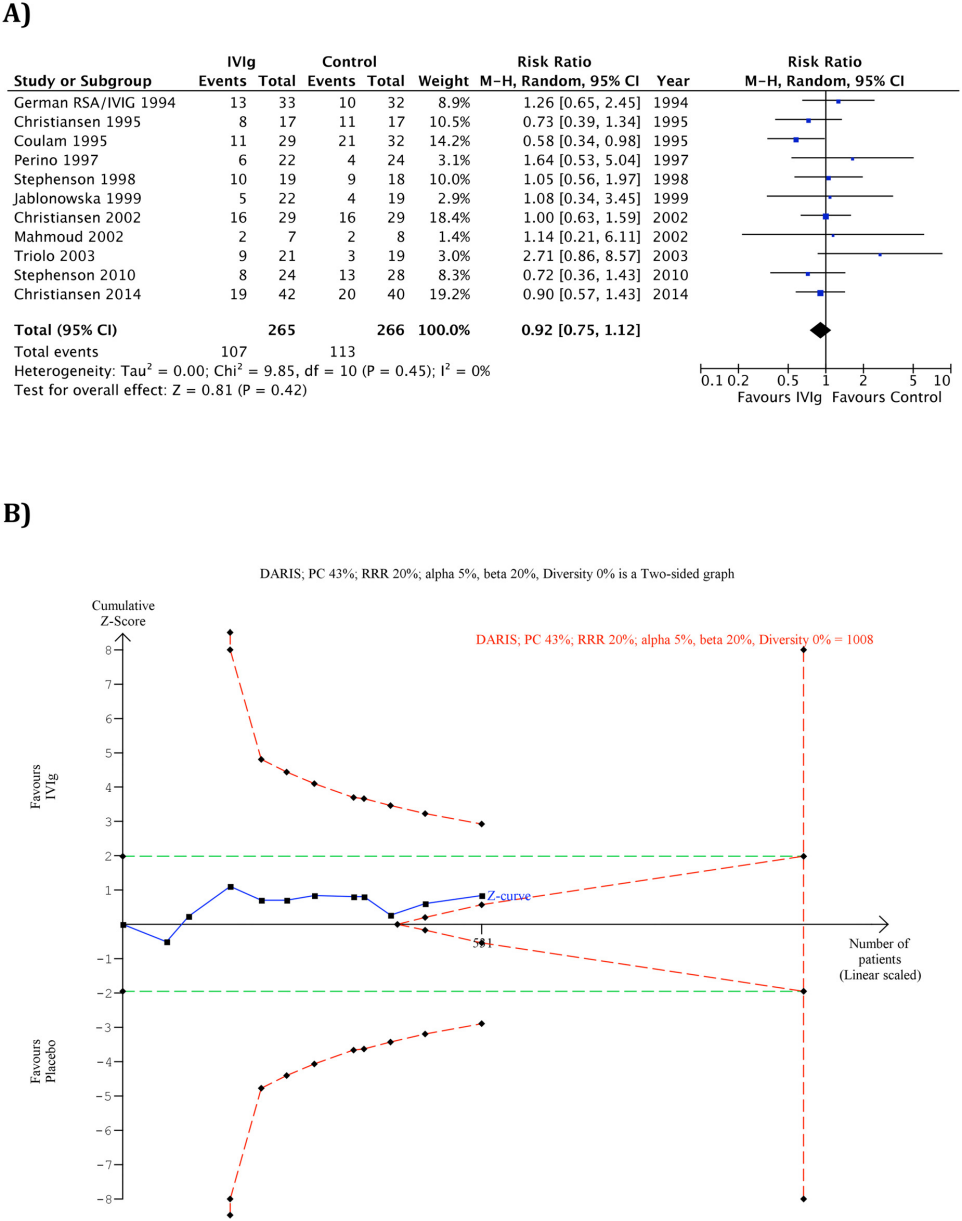
Meta-analysis for the outcome no live birth for all trials (A) and Trial sequential analysis for the outcome no live birth for all trials (B). A) Forest Plot of the meta-analysis for the outcome no live birth, B) The diversity-adjusted required information size (DARIS) of 1,008 patients was calculated on the basis of type I error of 5%, type II error of 20%, the control group event proportion (PC) of 43%, a relative risk reduction (RRR) of 20%, and the diversity (D^2^) 0% of the meta-analysis. The cumulative Z-curve does not cross the trial sequential monitoring boundaries for benefits, harms, or futility, and the required information size was not reached.

Fig 2A shows the meta-analysis for the outcome ‘no live birth’.

All 11 trials with a total of 531 women reported adequately on ´no live birth`. Overall, no significant difference in the number of ‘no live birth’ was found when IVIg was compared with placebo or treatment as usual (107/265 (40%) versus 113/266 (42%); RR: 0.92, 95% CI 0.75–1.12, p = 0.42). Funnel plot of the included 11 trials was symmetrical (not included). Fig 2B shows the TSA.

The cumulative Z-curve did not cross any of the trial sequential monitoring boundaries for benefit, harm, or futility and did not reach the required information size of 1,008 participants. The Z-curve was, however, very close to the futility boundary.

### The proportion of women experiencing serious adverse events (SAEs)

Eight trials reported on SAEs [5, 10–12, 14, 17, 38, 39]. The meta-analysis of the eight trials with a total of 381 women found overall no significant difference in the number of SAEs when IVIg was compared with placebo or treatment as usual (29/193 (15%) versus 27/188 (14%); RR: 1.06, 95% CI 0.67–1.67, p = 0.81). The description of SAEs included caesarean sections, premature rupture of membranes prior to gestational week 28, and hospitalisation due to infusion-related symptoms. None of the trials reported participants with death, HIV, or hepatitis. TSA showed that the cumulative Z-curve did not cross any of the monitoring boundaries for benefits, harms, or futility and the required information size of 4,410 participants was not accrued (data not shown).

The observational studies reported no SAEs among the women treated with IVIg [40–43].

### The proportion of live-born infants experiencing SAEs

Nine trials reported on infants experiencing SAEs [5, 9–12, 14, 15, 17, 38]. The meta-analysis of the nine trials with a total of 286 infants found overall no significant difference in the number of SAEs when IVIg was compared with placebo or treatment as usual (20/146 (14%) versus 17/140 (12%); RR: 1.18, 95% CI 0.58–2.42, p = 0.65). The description of SAEs included admission to neonatal care unit, congenital malformations, mental retardation, and death. TSA showed that the cumulative Z-curve did not cross any of the monitoring boundaries for benefits, harms, or futility, and the required information size of 5,251 participants was not obtained (data not shown).

### The proportion of women experiencing adverse events (AEs)

Nine trials with a total of 451 women reported on AEs [5, 10–15, 17, 38]. The meta-analyses indicate a higher frequency of AEs after IVIg treatment compared with placebo or treatment as usual (68/229 (30%) versus 42/222 (19%); random-effects model: RR: 1.55, 95% CI 0.89–2.69, p = 0.12, fixed-effects model: RR: 1.54, 95% CI 1.13–2.11, p = 0.006). The reporting on AEs varied widely between trials. The description of AEs included vaginal bleeding, rash, headache, fever, and itching.

The observational studies reported similar AEs as the randomised trials [40–43].

### The women’s quality of life

None of the trials reported on the women’s quality of life.

### Proportion of women who give live birth prematurely (<37 gestational weeks)

Ten trials with a total of 284 women found no significant difference in the number of premature live births when IVIg was compared with placebo or treatment as usual (12/141 (9%) versus 15/143 (10%); RR: 0.80, 95% CI 0.37–1.71, p = 0.56) [5, 10–15, 17, 38, 39].

### Sex of the infant

Only four trials with a total of 135 infants reported on sex of the infant [5, 14, 15, 17]. Overall, there were 64 boys and 71 girls and thereby overall slightly more girls (52.6%).

### The proportion of infants with low birth weight

Seven trials reported on low weight infants (<2500 g) [5, 10–14, 17]. The meta-analysis of the seven trials with a total of 238 infants found overall no significant difference in the number of low weight infants when IVIg was compared with placebo (10/120 (8%) versus 10/118 (8%); RR: 1.02, 95% CI 0.43–2.45, p = 0.96).

### The proportion of infants experiencing AEs

Only four trials reported on infants with AEs and found no AEs in both the IVIg and the placebo groups [5, 10, 14, 17].

### The infants’ quality of life

None of the trials reported on the infants’ quality of life.

### Sensitivity analyses

A total of four participants were lost to follow up. We performed ‘best-worst-case’ scenario and ‘worst-best-case’ scenario analyses [21], which did not change our results. We also performed a per-protocol analysis, which did not change our results.

As the trials were heterogeneous with regard to control intervention and initiation of the intervention, we further performed post-hoc sensitivity analyses where we excluded the trials that used treatment as usual as control intervention [38, 39], and where we excluded the trials that initiated IVIg intervention before pregnancy was obtained [9, 13, 15]. These sensitivity analyses did not change our results.

### Subgroup analyses

An overview of the data used in the subgroup analyses can be found in Table 3.

**Table 3:** Data sources for the subgroup analyses of the outcome ´no live birth`. Data sources for the subgroup analyses in relation to aggregate published data, individual patient data (IPD) from a trial where the randomisation was stratified according to this factor and IPD from a trial where the randomisation was not stratified according to this factor.

Subgroup analyses according to data	Women with primary compared to women with secondary RM	Women without lupus anticoagulant and/or IgG anticardiolipin compared to women with lupus anticoagulant and/or IgG anticardiolipin	Trials providing dosages of IVIg at or over the median compared to trials providing IVIg dosages below the median	Trials with ‘low risk of bias´ compared to trials with ‘high risk of bias´**	Women with two miscarriages compared to women with three miscarriages and to women with four or more miscarriages
**Data from all trial participants** *	245 participants:	408 participants:	531 participants:	531 participants;	140 participants:
	German RSA/IVIG 1994 [11]	Christiansen 1995 [10]	German RSA/IVIG 1994 [11]	German RSA/IVIG 1994 [11]	Christiansen 2002 [5]
	Perino 1997 [12]	Coulam 1995 [9]	Coulam 1995 [9]	Coulam 1995 [9]	Christiansen 2014 [17]
	Stephenson 2010 [15]	Perino 1997 [12]	Christiansen 1995 [10]	Christiansen 1995 [10]	
	Christiansen 2014 [17]	Stephenson 1998 [13]	Perino 1997 [12]	Perino 1997 [12]	
		Jablonowska 1999 [14]	Stephenson 1998 [13]	Stephenson 1998 [13]	
		Mahmoud 2002 [39]	Jablonowska 1999 [14]	Jablonowska 1999 [14]	
		Triolo 2003 [38]	Christiansen 2002 [5]	Christiansen 2002 [5]	
		Stephenson 2010 [15]	Mahmoud 2002 [39]	Mahmoud 2002 [39]	
		Christiansen 2014 [17]	Triolo 2003 [38]	Triolo 2003 [38]	
			Stephenson 2010 [15]	Stephenson 2010 [15]	
			Christiansen 2014 [17]	Christiansen 2014 [17]	
**Data from a subgroup of trial participants, stratified randomisation** ^†^	77 participants:				
	Stephenson 1998 [13].				
	Jablonowska 1999[14]				
**Data from a subgroup of trial participants, randomisation not stratified** ^‡^	80 participants:	58 participants:			158 participants:
	Christiansen 1995 [10]	Christiansen 2002 [5]			Christiansen 1995 [10]
	Christiansen 2002 [5]				Perino 1997 [12]
					Stephenson 1998[13]
					Jablonowska 1999[14]

* ‘Data from all trial participants’ includes data from a trial (both summary data and IPD), if all participants are in the same subgroup (e.g., when trial only includes women with secondary RM).

** Low risk of bias was determined based on selected domains (see Methods), which makes such trials vulnerable to bias from other domains.

^†^ ‘Data from a subgroup of trial participants, stratified randomisation’. This includes data from a subgroup of participants in a single trial (both summary data and IPD), where the randomisation is stratified according to the specific subgroup variable (e.g., separate randomisation strata for women with primary RM and secondary RM). Stratified randomisation secures that baseline characteristics are equally distributed in both intervention groups in both strata.

^‡^ ‘Data from a subgroup of trial participants, randomisation not stratified’. This includes data from a subgroup of participants in a single trial (both summary data and IPD), where the randomisation is not stratified according to the specific subgroup variable (e.g., one common allocation sequence for women with primary RM and secondary RM). When the randomisation is not stratified according to the specific subgroup variable, it is uncertain if baseline characteristics are equally distributed in both intervention groups in both subgroups. This means that subgroup data may resemble observational data. Thus, results based on this data have reduced inferential power.

#### Primary compared to secondary RM

**Fig 3:**
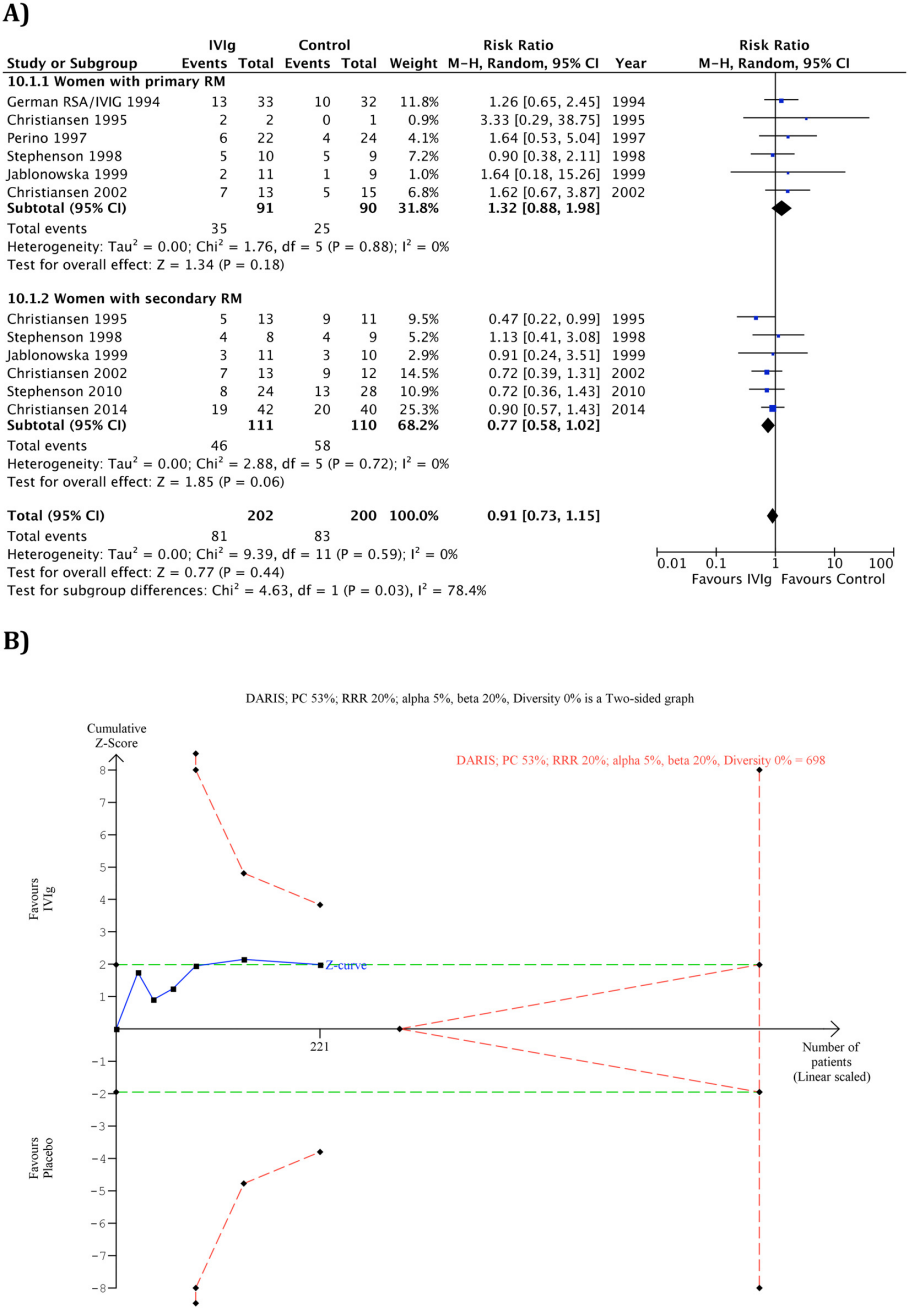
Meta-analysis for the subgroup analysis for women with primary RM compared to secondary RM (A) and Trial sequential analysis for women with secondary RM (B). A) Forest Plot for the outcome no live birth, B) The diversity-adjusted required information size (DARIS) of 698 patients was calculated on the basis of type I error of 5%, type II error of 20%, the control group event proportion (PC) of 53%, a relative risk reduction (RRR) of 20%, and the diversity (D^2^) 0% of the meta-analysis. The cumulative Z-curve does not cross trial sequential monitoring boundaries for benefits, harms, or futility, and the required information size was not reached.

The subgroup analysis for women with primary RM compared to secondary RM is illustrated in Fig 3A.

A statistically significantly test of interaction for outcome ´no live birth`(p = 0.03) was found, indicating that IVIg may have different effects in women with primary RM (35/91 (38%) versus 25/90 (28%); RR: 1.32, 95% CI 0.88–1.98, p = 0.18) compared to women with secondary RM (46/111 (41%) versus 58/110 (53%); RR: 0.77, 95% CI 0.58–1.02, p = 0.06). Fig 3B shows TSA of IVIg compared with placebo or treatment as usual in participants with secondary RM. The cumulative Z-curve did not cross any of the monitoring boundaries for benefits, harms, or futility, and the required information size of 698 was not reached.

#### Low compared with high IVIg dosage

The planned total median dosage of IVIg provided from before conception to gestational week 10 in all trials was calculated to be approximately 84 g. Test for subgroup differences for trials providing dosages of IVIg at or over this median dose (RR for ´no live birth`: 0.85, 95% CI 0.64–1.12 p = 0.25) compared to trials providing IVIg doses below the median dose (RR for ´no live birth`: 1.19, 95% CI 0.81–1.75, p = 0.38) was not statistically significantly different (test of interaction, p = 0.17). We post-hoc conducted a meta-analysis in order to evaluate if there was a relation between IVIg dosage and the proportion of women experiencing AEs. The meta-analyses indicate a higher frequency of AEs after IVIg treatment at or over the median dosage (RR 2.33, 95% CI 1.00–5.46, p = 0.05) compared to IVIg treatment below the median dosage (RR 0.85, 95% CI 0.46–1.58, p = 0.61), test for subgroup differences p = 0.06 (random effect model) and p = 0.02 (fixed effect model).

#### Other subgroup analyses

Tests for subgroup difference for trials with low risk of bias regarding all domains or regarding the selected domains: allocation sequence, allocation concealment, and blinding of participants and treatment providers compared to trials with ‘high risk of bias’ (p = 0.75); for patients without lupus anticoagulant and/or IgG anticardiolipin compared to patients with lupus anticoagulant and/or IgG anticardiolipin (p = 0.60); for participants with two miscarriages compared to participants with three miscarriages and to participants with four or more miscarriages (p = 0.90) were not statistically significantly different for the outcome ‘no live birth’.

## Discussion

### Main findings

We identified 11 trials accessing the effect of IVIg in women with RM. Only one trial was classified as ‘low risk of bias’ for all domains, and 10 trials as ‘high risk of bias’. We identified four out of these 10 trials that could be considered trials with ‘low risk of bias’ for selected domains according to our protocol [21]. Such trials can of course be biased due to other domains. We were able to obtain IPD from only six trials. Overall, treatment with IVIg did not reduce the risk for the outcome ‘no live birth’ in women with RM when compared with placebo or treatment as usual. TSA on the outcome ‘no live birth’ showed that the cumulative Z-curve did not cross the monitoring boundaries for benefits, harms, or futility and the required information size of 1,008 participants was not reached. Accordingly, we cannot interpret absence of evidence as evidence of absence of intervention effect. The Z-curve was, however, very close to the futility boundary. When the futility boundary is crossed, the test statistic is so low that the likelihood of a statistically significant intervention effect being found if and when the required information size is reached becomes equivalent to the risk of type II error [31]. When the Z-curve is so close to the futility boundary, it is possible that a new trial will cross the futility boundaries. However, we may be quite far from the futility boundaries based on a smaller intervention effect than a relative risk reduction of 20% we used.

The meta-analysis of the proportion of women with AE indicates a greater frequency of AE after IVIg treatment compared with placebo or treatment as usual. The children, however, did not seem to suffer from the intervention, although evidence was sparse.

Test for subgroup differences showed that there was a statistically significant difference in the effect of IVIg in women with primary RM compared to women with secondary RM for the outcome ‘no live birth’. This may indicate that a potential beneficial effect of IVIg may be limited to women with secondary RM. However, TSA of the trials only including women with secondary RM showed that the cumulative Z-curve did not cross any of the trial sequential monitoring boundaries for benefits, harms, or futility, and the required information size of 698 participants was not obtained (so far only 221 women with secondary RM have been randomised), meaning that more trials are needed before any conclusions can be made.

The theoretical explanation for the observed difference in the effect of IVIg in women with primary compared to women with secondary RM may be that immunological disturbances seem to play a larger role in women with secondary RM. It has been reported that there is a higher prevalence of the immunological high responder HLA allele HLA-DRB1*03 in women with secondary RM compared to controls [45], that maternal carriage of other HLA class II alleles associated with immunity against male-specific minor histocompatibility (HY-) antigens results in a reduced live birth proportion in secondary RM patients with a firstborn boy [46], and that plasma levels of the proinflammatory and potentially harmful cytokine TNF-α are increased in patients with secondary RM relative to those with primary RM [47].

### Strengths and limitations

Our systematic review has a series of strengths. We conducted the review according to the recommendations stated in The Cochrane Handbook for Systematic Review of Interventions [22]. Furthermore, we followed our published protocol [21] with predefined participants, interventions, outcomes, and comparisons in order to avoid biases in the review process. We performed an extensive literature search to identify relevant studies based on our predefined inclusion criteria.

We contacted all original authors in order to obtain IPD but were only successful in getting data from four trials and in addition some IPD data could be extracted from the publication of two other trials. To avoid bias, we supplemented the available IPD with aggregate data from those trials, were IPD were not available, which makes the analyses more relevant. We placed participants into subgroups within the trials using IPD. In three instances (women with primary RM compared to women with secondary RM [5, 10]; women without lupus anticoagulant and/or IgG anticardiolipin compared to women with lupus anticoagulant and/or IgG anticardiolipin [5]; and women with two miscarriages compared to women with three miscarriages and to women with four or more miscarriages [10, 12–14], the original trials had not stratified the randomisation according to the subgroup variable. This means that data will likely resemble more observational data, as the balance obtained by randomisation no longer can be guaranteed. Results from these subgroup analyses must therefore be interpreted with caution.

We conducted meta-analyses based on intention-to-treat principles, whereby all participants randomised in each trial were included in the analyses. It also has to be noted that we due to IPD were able to analyse almost all the predefined outcomes except quality of life.

We did not plan to assess our review with the Grading of Recommendations Assessment, Development and Evaluation system (GRADE) [48]. With GRADE we would have downgraded the evidence to ‘low quality’ or ‘very low quality’ according to the risk of bias, risk of imprecision, and risk of indirectness. We found low heterogeneity and no evidence of publication bias. Our review’s findings and interpretations suffer from limitations ensuring from the low quality of the randomised trials included in our review. After risk of bias assessment, all trials except one were considered with high risk of bias. To try to avoid potential bias from the trials with ‘high risk of bias’, we performed a predefined subgroup analysis where trials with ‘low risk of bias’ (that is low risk of bias in only selected domains) were compared to trials with ‘high risk of bias’. We were unable to find a difference in the effects of IVIg in relation to the trials’ risk of bias. Our analyses for risks of random errors showed lack of sufficient information.

### Interpretation

Three prior meta-analyses on the topic were carried out by Wong et al. [16], Hutton et al. [49], and Ata et al. [50]. Hutton et al. found a significant treatment effect of IVIg in women with secondary RM whereas Wong et al. and Ata et al. concluded that there was no significant treatment effect of IVIg in women with RM.

In the analysis by Ata et al. two trials by Christiansen et al. (1995, 2002) comprising 92 patients were completely excluded, the main argumentation being that the trials included patients with antiphospholipid antibodies. This argument was rejected as none of the participants in the Christiansen et al. 1995 trial were antiphospholipid positive and in the Christiansen et al. 2002 trial only 5% were positive for these antibodies [51].

The Cochrane systematic review with meta-analysis by Wong et al. was updated in February 2014 [16]. No new trials of IVIg were included compared with the update in 2006 although a relevant new trial was published already in 2010 [15], a trial which was, however, included in the Ata et al. meta-analysis. The Cochrane review only includes one outcome (live birth after 20 weeks´ of gestation in all patients) and a single subgroup analysis (trials reporting intention-to—treat analysis).

Our systematic review included more trials than the existing meta-analyses and more patients thus decreasing possible bias in the available evidence. Two of the reviews had according to our knowledge no published protocols prior to the conduct of the reviews [49, 50]. In order to avoid bias in the review process and to ensure transparency we prepared a detailed and comprehensive protocol which we made publically available [21].

In addition, we conducted TSA, which has not been conducted in any of the previous meta-analyses [16, 49, 50]. TSA deals with the risk of random error and is important to assess how far we are from having reached the required information size in order to conclude the true effects of a specific intervention. Conclusions made using TSA show the potential to be more reliable than those using traditional meta-analysis techniques [31].

Moreover, we tried to obtain IPD. IPD meta-analyses use a much richer dataset and thus have greater statistical power than conventional meta-analysis. It is also an advantageous methodological approach when subgroup analyses are needed. Subgroup analysis is very clinically relevant since it allows a more personalised treatment.

## Conclusion

Based on our results, we have insufficient evidence to recommend or refute IVIg for women with RM. Treatment with IVIg compared with placebo seems to increase the risk of adverse events. Subgroup analysis suggests that women with secondary RM seemed most likely to obtain a potential beneficial effect of IVIg, however, trial sequential analysis showed that insufficient information is presently accrued. IVIg should therefore only be administered in randomised clinical trials, clearly explaining the potential risks to participants. According to our subgroup analyses, women with secondary RM are the group, which most likely should be included in such trials.

## Supporting Information

S1 File Characteristics of excluded studies. (DOCX)

S2 File Search strategies I. (DOC)

S3 File Search strategies II. (DOC)

S4 File PRISMA Checklist. (DOC)

S5 File Data set. (RM5)
