# Supplementary material for: The Effects of Intravenous Immunoglobulins in Women with Recurrent Miscarriages: A Systematic Review of Randomised Trials with Meta-Analyses and Trial Sequential Analyses Including Individual Patient Data
Source: PLoS One. 2015 Oct 30;10(10):e0141588. doi: 10.1371/journal.pone.0141588 (PMC4627734; doi:10.1371/journal.pone.0141588)
Supplement: S1 File — (DOCX) [file pone.0141588.s001.docx]

**Characteristics of excluded studies**

| Practice Committee of the American Society for Reproductive Medine, 2006 | Committee opinion. Not a randomized controlled trial. |
| --- | --- |
| Ata, Reply, 2011 | Letter to the editor, authors reply. Not a randomized trial. Refer to Ata 2011. |
| Ata, 2011 | A systematic review. Not a randomised trial. |
| Branch, 2001 | A review. Not a randomised trial. |
| Branch, 2000 | The included patients did not have recurrent miscarriage. |
| Carp, 2001 | Controlled, but not randomised. |
| Christiansen, 2011 | Letter to editor. Not a randomised trial. Refer to Ata 2011. |
| Christiansen, 1992 | Not a randomised trial. |
| Collins, 1994 | Not a randomised trial, but a review. Not exclusively IVIg intervention. |
| Coulam, 1992 | Not a randomised trial. |
| Coulamcacc | Duplicate |
| Daya, 1999 | A systematic review with individual patient data. Not a randomised trial. |
| De Placido | Participants not pregnant at randomisation |
| Hutton, 2006 | A systematic review. Not a randomised trial. |
| Illeni, 1994 | A randomised trial comparing expectant management versus immunotherapy with paternal leukocytes. |
| Kiprov, 1996 | Not a randomised trial. |
| Kuhn, 1996 | Review, not a randomised trial. |
| Kwon, 2012a | A trial of women with endometriosis without recurrent miscarriage. |
| Kwon, 2012b | Duplicate |
| Marzusch, 1996 | Not a randomised trial |
| Moraru, 2012 | Not a randomised trial. |
| Orange, 2006 | A review, not a randomised trial. |
| Practice Committee of the American Society for Reproductive Medine, 2004 | Committee opinion. Not a randomised trial. |
| Practice Committee of the American Society for Reproductive Medine, 2004 | Duplicate. |
| Practice Committee of the American Society for Reproductive Medine, 2006 | Duplicate. |
| Razaei, 2011 | Review, not a randomised trial. |
| Schneider, 1998 | Not a randomised trial. |
| Stein, 2000 | Not a randomised trial. |
| Stephenson, 2000 | The included patients did not have recurrent miscarriages, but a study of women with repeated unexplained IVF failure. |
| Stricker, 2000 | Not a randomised trial. |
| Stricker, 2005 | Not a randomised trial. |
| Stricker, 2002 | A review, not a randomised trial. |
| Sun, 2010 | Not a randomised trial. |
| Winger, 2011 | A study investigating IVIg treatment in subfertile women undergoing IVF. Not a randomised trial. |
| Zingsem…, 1994 | Duplicate |
