# Supplementary material for: The Effects of Intravenous Immunoglobulins in Women with Recurrent Miscarriages: A Systematic Review of Randomised Trials with Meta-Analyses and Trial Sequential Analyses Including Individual Patient Data
Source: PLoS One. 2015 Oct 30;10(10):e0141588. doi: 10.1371/journal.pone.0141588 (PMC4627734; doi:10.1371/journal.pone.0141588)
Supplement: S3 File — (DOC) [file pone.0141588.s003.doc]

**Search strategies for**

**IvIg for recurrent miscarriages**

**(P Egerup)**

**Searches performed 2 April 2014**

**Number of references identified 814 references**

**Number of duplicates removed 253 references**

**Final number of references in list 561 references**

**Batch name:** 140403_P Egerup_IvIg for recurrent miscarriages

**Cochrane Central Register of Controlled Trials (CENTRAL)(Issue 1 of 12, 2014) (86 hits in CENTRAL)**

#1 MeSH descriptor: [Immunotherapy] explode all trees

#2 MeSH descriptor: [Immunoglobulins, Intravenous] explode all trees

#3 (immunotherap* or immunisation or ((immunoglobulin* or Ig) and (intravenous or IV)))

#4 #1 or #2 or #3

#5 MeSH descriptor: [Fetal Death] explode all trees

#6 MeSH descriptor: [Abortion, Spontaneous] explode all trees

#7 ((f?etal and (death or loss)) or abortion* or miscarriage*)

#8 #5 or #6 or #7

#9 #4 and #8

**MEDLINE (Ovid SP)(1946 to April 2014)(190 hits)**

1. exp Immunotherapy/

2. exp Immunoglobulins, Intravenous/

3. (immunotherap* or immunisation or ((immunoglobulin* or Ig) and (intravenous or IV))).mp. [mp=title, abstract, original title, name of substance word, subject heading word, keyword heading word, protocol supplementary concept word, rare disease supplementary concept word, unique identifier]

4. 1 or 2 or 3

5. exp Fetal Death/

6. exp Abortion, Spontaneous/

7. ((f?etal and (death or loss)) or abortion* or miscarriage*).mp. [mp=title, abstract, original title, name of substance word, subject heading word, keyword heading word, protocol supplementary concept word, rare disease supplementary concept word, unique identifier]

8. 5 or 6 or 7

9. 4 and 8

10. (random* or blind* or placebo* or meta-analys*).mp. [mp=title, abstract, original title, name of substance word, subject heading word, keyword heading word, protocol supplementary concept word, rare disease supplementary concept word, unique identifier]

11. 9 and 10

**EMBASE (Ovid SP)(1974 to April 2014)(378 hits)**

1. exp immunotherapy/

2. exp immunoglobulin/

3. (immunotherap* or immunisation or ((immunoglobulin* or Ig) and (intravenous or IV))).mp. [mp=title, abstract, subject headings, heading word, drug trade name, original title, device manufacturer, drug manufacturer, device trade name, keyword]

4. 1 or 2 or 3

5. exp fetus death/

6. exp abortion/

7. ((f?etal and (death or loss)) or abortion* or miscarriage*).mp. [mp=title, abstract, subject headings, heading word, drug trade name, original title, device manufacturer, drug manufacturer, device trade name, keyword]

8. 5 or 6 or 7

9. 4 and 8

10. (random* or blind* or placebo* or meta-analys*).mp. [mp=title, abstract, subject headings, heading word, drug trade name, original title, device manufacturer, drug manufacturer, device trade name, keyword]

11. 9 and 10

**Science Citation Index Expanded (1900 to April 2014)(160 hits)**

#5 160 #4 AND #3

#4 1,212,637 TS=(random* or blind* or placebo* or meta-analys*)

#3 857 #2 AND #1

#2 41,371 TS=((f?etal and (death or loss)) or abortion* or miscarriage*)

#1 149,976 TS=(immunotherap* or immunisation or ((immunoglobulin* or Ig) and (intravenous or IV)))
